# Using mHealth for Primary Prevention of Dementia: A Proof-of-Concept Study on Usage Patterns, Appreciation, and Beliefs and Attitudes Regarding Prevention

**DOI:** 10.3233/JAD-230225

**Published:** 2023-08-01

**Authors:** Irene Heger, Kay Deckers, Marjolein de Vugt, Frans Verhey, Anke Oenema, Martin van Boxtel, Sebastian Köhler

**Affiliations:** a Alzheimer Centrum Limburg, School for Mental Health and Neuroscience, Maastricht University, Maastricht, The Netherlands; b Department of Health Promotion, CAPHRI Care and Public Health Research Institute Maastricht, NUTRIM School of Nutrition and Translational Research in Metabolism, Maastricht University, Maastricht, The Netherlands

**Keywords:** Alzheimer’s disease, awareness, dementia, mobile applications, primary prevention, protective factors, public health, risk assessment, risk factors, risk reduction behavior

## Abstract

**Background:**

Health- and lifestyle factors account for a substantial part of all dementia cases, which opens the opportunity for primary prevention. However, the required behavioral change is complex and involves targeting multiple risk factors. mHealth interventions can potentially contribute to improving motivation in a low-cost and scalable way.

**Objective:**

To explore usage patterns, appreciation, and beliefs and attitudes regarding dementia risk reduction during the use of the MyBraincoach mobile app.

**Methods:**

Participants were community-dwelling middle-aged adults from the Netherlands and used either the standard (education) or extended (education+motivational triggers) app version for three months. Two panel studies were combined in this paper. Chi-square tests, *t*-tests and linear mixed models were used, adjusted for age, sex, and education.

**Results:**

Of all participants (*n* = 299, 50.2% male), 167 (55.9%) had installed the app. The most reported reason for non-use was technical problems (47%). Those who used the app were at baseline already more positive about dementia risk reduction than those who did not use the app. Of all users who completed the evaluation (*n* = 102), 78.4% (*n* = 80) stated that the app provided a positive approach towards brain health and 80.4% (*n* = 82) felt better informed. Younger (<60y) and lower educated participants evaluated the app most positively.

**Conclusion:**

Usage of the app was low, but users showed more positive beliefs and attitudes regarding dementia risk reduction. Most users evaluated the app positively and stated to have gained knowledge on the topic. Improving the use of the app must keep high priority in future studies.

## INTRODUCTION

There is increasing evidence that dementia is associated with modifiable risk and protective factors, such as smoking, obesity, and physical inactivity [1–4], which opens up the opportunity for primary prevention. This is underscored by the 2020 report of the Lancet Commission on Dementia Prevention, Intervention, and Care that concluded that a total of 12 modifiable factors account for around 40% of the people with dementia worldwide [2]. Most of these risk factors were also incorporated in the dementia risk reduction guidelines of the World Health Organization [5].

To optimize this potential of primary prevention of dementia, applying the existing knowledge to prevention strategies is essential. However, awareness in the general population about the relationship between lifestyle and dementia risk is low and raising awareness is crucial for the required behavioral change [6, 7]. In addition, questions remain how to raise motivation. Several social and cognitive theories show that behavior is influenced by underlying beliefs and attitudes [8]. For instance, the Theory of Planned Behavior assumes that constructs such as beliefs (e.g., about the consequence of the behavior and expectations from others), attitudes (positive of negative value of the behavior), and subjective norms (social pressure to engage in the behavior) influence intention to perform a certain behavior. Intention and perceived behavioral control (i.e., perceived ability to perform a behavior), in turn, determine actual behavior [8]. Changing behavior is thus complex. Further, adapting the lifestyle for a long-term cause such as dementia prevention makes it even more complex.

Public health initiatives could potentially motivate people to adopt a brain-healthy lifestyle and thereby lower the worldwide burden of dementia [2, 9–12]. Using mHealth for this purpose offers several advantages over traditional, face-to-face public health methods. For example, the larger reach (over 92% of the Dutch population was in the possession of a smartphone in 2019 [13]), the easy accessibility, increased adherence, low costs [14, 15], and consequently potentially less socioeconomic disparity [16]. In fact, our own survey from 2019 showed that 81% of the general population 40–75 years old would like to or would consider using an app to learn more about brain health [7].

Therefore, this proof-of-concept study investigates a mobile app focused on education and motivation for dementia risk reduction in middle-aged, cognitively healthy community-dwelling participants from the Netherlands. We aim to 1) explore the usage pattern and user appreciation and 2) measure beliefs and attitudes regarding motivation to modify dementia risk behaviors.

## METHODS

### Study design

This paper combined two panel studies regarding the MyBraincoach (Dutch: ‘MijnBreincoach’) app [17] that took place in May to July 2018 and in October to December 2019. In the 2018 study, participants used the standard version of the app (see Methods section, *Standard version of the MyBraincoach mobile app*). After completion of this study, an adapted version of the app was developed that had extended functions (see Methods section, *Extended version of the MyBraincoach mobile app*). During the 2019 study, participants were either randomized to the group that used the standard version, or to the group that used the extended version. For this paper, data from both studies were combined for a larger sample size and to allow comparison between the two versions of the app. All methods were carried out in accordance with relevant guidelines and regulations and in accordance with the Helsinki Declaration. The Ethics Review Committee Psychology and Neuroscience (ERCPN) of Maastricht University approved the 2018 study (ERCPN- 185_01_11_2017) and the 2019 study (reference number ERCPN-208_08_05_2019). All participants received written information about the study and signed an online informed consent form. An adaptation of this study has been published as a PhD thesis and we have approval to republish the data [18].

### Participants

Eligible for this study were community-dwelling, middle-aged (40 to 75 years) individuals without dementia (self-report), and with access to a smartphone with Internet connection, living in the Netherlands. The study aimed to include 100 participants in the 2018-study and 200 participants (*n* = 100 standard app, *n* = 100 extended app) in the 2019-study.

### Procedure

**Fig. 1:**
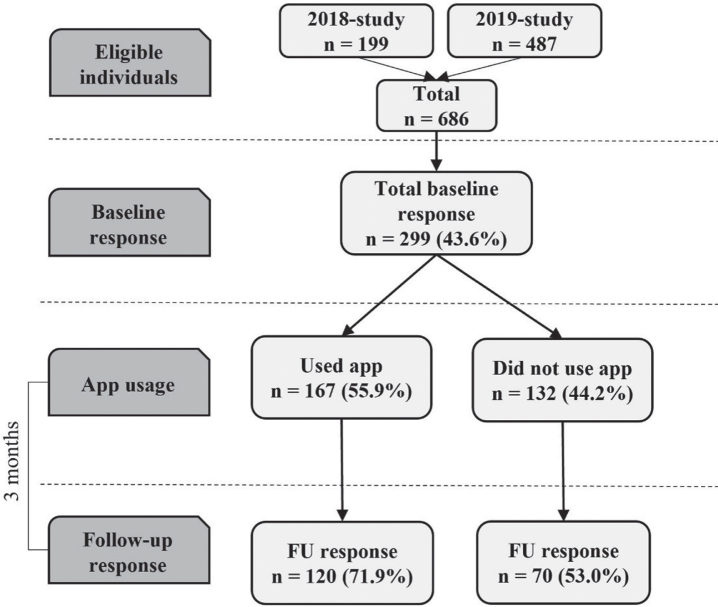
Flowchart of the recruitment process, app use, and survey response. Recruitment via: eligible individuals from responder samples of surveys evaluating a health campaign and additional recruitment in the 2019-study via advertisement on website Dutch Brain Foundation and newsletter of a large health care institution in the south of the Netherlands.

Participants were sampled from responders of previous dementia risk awareness surveys [7, 19] who agreed to participate in future research. Figure 1 provides a flowchart of the recruitment process, app use, and survey response. All eligible individuals were contacted via e-mail, with information on the study (including information on length of time of the survey and anonymous data storage) and a hyperlink to the online informed consent form and baseline questionnaire using Lime Survey software (see Methods section, *Measures*). After completion of the baseline questionnaire, participants received an email with detailed information on how to install the app. They were requested to use the app daily for the next twelve weeks. Participants could email or call the research team with questions and remarks concerning the installation process and use of the app. Every two weeks, a reminder email was sent to all participants encouraging them to use the app. Halfway through the intervention period (after 6 weeks), a personal email was sent to all participants who had not yet installed the app. After the intervention period of 12 weeks, all participants received an e-mail with a personal and secured hyperlink to the online follow-up questionnaire. All participants who completed the study received a gift card of 20 Euro. Data was anonymized, stored on a protected server of the research setting and access was only granted to the authors that performed the statistical analyses (IH, SK).

### Standard version of the MyBraincoach mobile app

#### Key functionalities

**Fig. 2:**
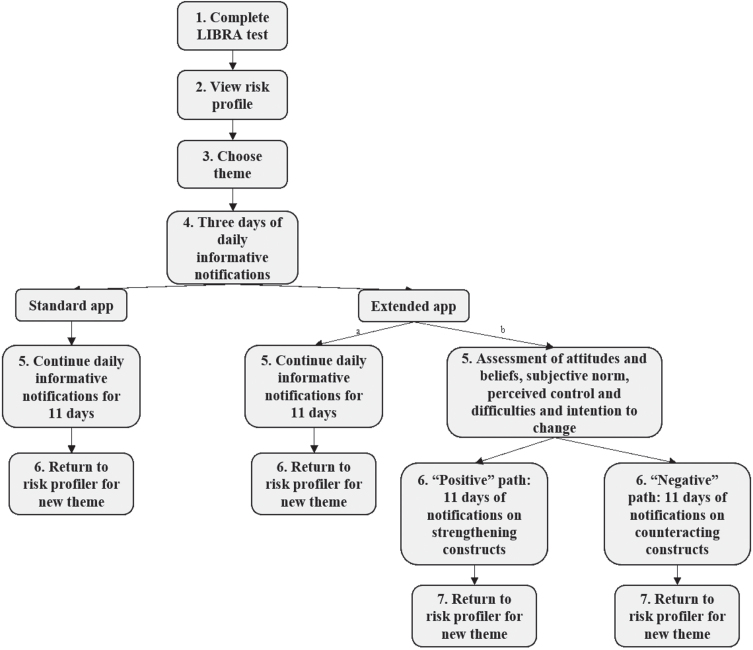
Graphic overview of the functionalities of the standard and extended MyBraincoach app. ^a^Path in the extended app when choosing the (not-directly modifiable) factors coronary heart disease, chronic kidney disease, diabetes, cholesterol, hypertension, obesity, or depression. ^b^Path in the extended app when choosing the (directly modifiable) factors alcohol consumption, physical inactivity, smoking, diet, or cognitive activity. LIBRA, LIfestyle for BRAin health index.

**Fig. 3:**
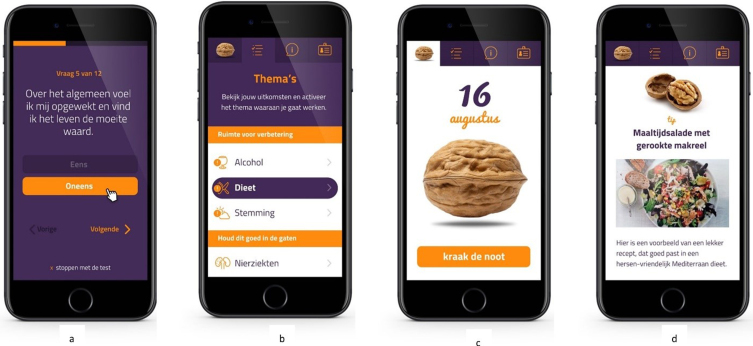
User interface MyBraincoach app. a) Completing the LIBRA test; b) Personal brain health profile; c) Presentation of daily notification (“Walnut of the day”); d) Content of daily notification (screenshot from LIBRA theme diet: advice in the form of a brain-healthy recipe). LIBRA, LIfestyle for BRAin health index.

The standard app version was designed to give people insight into their personal dementia risk profile and to identify room-for-improvement, based on the validated LIfestyle for BRAin health (LIBRA) score [20]. The LIBRA score, that was already used as a framework for a dementia risk reduction intervention discussed in a recent review, consists of 12 modifiable risk and protective factors for cognitive decline and dementia and has been extensively shown to predict dementia risk [21–23] and change in multimodal lifestyle interventions [24, 25]. Table 1 provides an overview of all LIBRA factors and the instruments and cut-offs used. For instance, the LIBRA factor high cognitive activity was operationalized by the Cognitive Reserve Index questionnaire (CRIq), which assesses education, working activity and leisure time [26]. Figure 2 gives an overview of the different functionalities within the app, and Fig. 3 provides accompanying illustrative screenshots of the user interface. First, users completed a comprehensive assessment of the 12 LIBRA factors (see step 1 in Figs. 2, and 3a). After completion, a personal profile was created (See step 2 in Figs. 2, and 3b) that gave people insight into identified areas of healthy behavior (to promote maintenance), identified areas of unhealthy behavior (to promote change) and identified chronic vascular/metabolic conditions (to promote appropriate management). Next, users were invited to choose a lifestyle topic or health condition of interest to work on (step 3 in Fig. 2). They then received a short daily notification (the “Walnut of the day”; 14 in total) which contained a short text message providing either educational information, a brain-health advice, a quiz item, or a behavioral challenge in a pseudo-random fashion (See steps 4 and 5 in Figs. 2, and 3b,c). If the daily notification was not opened, its content was presented again the next day. Users could change their topic of interest at any time (step 6 in Fig. 2).

**Table 1:** Overview of the questions, instruments, and cut-offs for the 12 LIBRA factors

	Questions/instruments	Cut-off
Low-to-moderate alcohol consumption	What is your daily, average alcohol consumption?	I don’t drink alcohol (–1.0)I drink up to 1 glass a day (–1.0)I drink more than 1 glass a day (0)
Coronary heart disease	Has your doctor ever told you that you have a heart- or blood vessel condition (including heart attack, angina (chest pain), heart failure, stroke or TIA’s)?	Yes (+1.0)No (0)I don’t know (0)
Physical inactivity	European Prospective Investigation into Cancer and Nutrition (EPIC) Physical Activity Questionnaire [27, 28]	Inactive or moderately inactive (+1.1)Moderately active or active (0)
Chronic kidney disease	Has your doctor ever told you that you have chronic kidney disease?	Yes (+1.1)No (0)I don’t know (0)
Diabetes	Has your doctor ever told you that you have diabetes?	Yes (+1.3)No (0)I don’t know (0)
Cholesterol	Has your doctor ever told you that your cholesterol is too high?	Yes (+1.4)No (0)I don’t know (0)
Smoking	Do you smoke?	Yes (+1.5)No (0)
Obesity	How long are you (in centimeters)?How much do you weigh? (Round off on whole kilograms. *Compute body mass index (BMI)* = *weight (kg)/length (m^2^)*	BMI≥30 (+1.6)BMI < 30 (0)
Hypertension	Has your doctor ever told you that you have high blood pressure?	Yes (+1.6)No (0)I don’t know (0)
Healthy diet/ Mediterranean diet	Mediterranean Diet Adherence Screener (MEDAS) [29]	MEDAS total score≤8 (–1.7)MEDAS total score < 8 (0)
Depression	Patient Health Questionnaire (PHQ-9) [30]	PHQ-9 score > 9 (+2.1)PHQ-9 score≤9 (0)
High cognitive activity	Cognitive Reserve Index questionnaire (CRIq) [26]	CRIq total score≥130 (–3.2)CRIq total score < 130 (0)

LIBRA, LIfestyle for BRAin health index; TIA, transient ischemic attack; BMI, body mass index.

### Extended version of the MyBraincoach mobile app

#### Key functionalities

The additional aim of the extended version of the app was to guide participants further in the process of behavior change, by addressing beliefs and attitudes about lifestyle and health behavior changes for dementia risk reduction. This additional feature was only integrated for the five LIBRA factors that were directly modifiable through individual lifestyle change (i.e., healthy diet, physical activity, cognitive activity, smoking, alcohol use; see step 5b of Fig. 2). The Determinants of Lifestyle Behavior Questionnaire (DLBQ) [31], an instrument based on Ajzen’s Theory of Planned Behavior [8, 32], was used to assess the determinants attitudes, subjective norms, perceived control and difficulties and intention to change for physical activity, dietary behavior, and smoking and to provide tailored advice. Similar items were constructed to also assess determinants of the LIBRA factors “cognitive activity” and “alcohol use” (e.g., perceived difficulties: “I find it difficult not to smoke under stressful circumstances” was changed into “I find it difficult to drink less alcohol under stressful circumstances”). The daily notifications for the other seven LIBRA factors were the same as the standard version of the app (see step 5a of Fig. 2). Based on a custom-made cut-off score, users were classified in a positive or negative path. In the next eleven days, participants received messages to counteract negative and strengthen positive constructs. The messages contained text, but also active elements, such as writing down (dis)advantages of changing behavior, goals, and action plans to prevent relapse to old behavior. Similar to the standard app, each topic consisted of 14 daily notifications.

### Development and testing of the apps

In the developmental phase of both versions of the app, scrum sessions were organized with the software partners, important stakeholders, and end-users in which the framework of the app was developed. The daily notifications were custom-made and derived from knowledge and advice from websites and flyers of evidence-based resources (e.g., the Dutch Heart Foundation, the Netherlands Nutrition Centre, Dutch Brain Foundation). After the research team had created 14 short notification messages based on these resources for each LIBRA theme, all content was checked by content experts on accuracy, completeness, and readability. Further, we tested the acceptance and look-and-feel of both versions of the app in a client panel of the Alzheimer Centre Limburg of people with dementia, their caregivers and people with a specific interest in the topic of brain health and dementia. A final pilot study (*n* = 20) was conducted in which participants (from the target population) tested the apps for two consecutive weeks, to identify technical issues and survey items that needed further modification.

### Measures

This study made use of usage tracking data to assess the usage pattern and of online questionnaires to assess beliefs and attitudes regarding behavioral change for dementia risk reduction, reasons for non-use and drop-out, and appreciation ofthe app.

### Usage tracking data

An export of usage tracking data was used to assess the usage pattern of all participants. This provided data for each individual participant on the personal risk profile, lifestyle topics chosen to receive daily notifications, the number of daily notifications received and the number of days the app was used.

### Online questionnaires

Participants completed an online questionnaire (see Fig. 1, baseline and follow-up response). The online questionnaire at baseline included items on demographics (i.e., age, sex, educational level, marital status), mobile operating system (iOS or Android) and beliefs and attitudes regarding behavioral change for dementia risk reduction by means of the Motivation to Change Lifestyle and Health Behaviors for Dementia Risk Reduction (MCLHB-DRR) Scale [33]. This 27-item scale assesses seven factors that reflect dimensions of the Health Belief Model, i.e., perceived susceptibility (4 items), perceived severity (5 items), perceived benefits (4 items), perceived barriers (4 items), cues to action (4 items), general health motivation (4 items), and self-efficacy (2 items). The Health Belief Model and the MCLHB-DRR Scale are explained in more depth in the paper describing the development of the scale [12]. This study used the 23-item Dutch validated version of the MCLHB-DRR as a primary outcome [34]. The online follow-up questionnaire after the 12-weeks intervention period included the MCLHB-DRR Scale again, plus a custom questionnaire on user appreciation. Participants who reported not to have installed the app completed statements on reasons why, and participants who reported to have used the app completed statements on appreciation, accompanying illustrative screenshots of the app. Users of the 2019 study who used the extended app version answered extra statements evaluating the additional features of this app. In answering the statements, participants were asked to what extent they agreed or disagreed on a 5-point Likert scale ranging from “strongly agree” to “strongly disagree”. This questionnaire was developed in consultation with the department of Health Promotion of Maastricht University and tested on understandability in two pilot studies in responders from the same target population.

### Statistical analyses

For the purpose of this study, the 2018 and 2019 studies were pooled as one sample in the analyses, while additional analyses stratified for study year and version of the app. Due to incomplete follow-up responses, the number of people included in the analyses may differ from the total number of people.

For analyses on usage pattern of the app, we used chi-squared tests and t-tests and included data from the usage-tracking system of all participants that used the app (see Fig. 1, app usage). Analyses on reasons for non-use or drop-out, beliefs and attitudes related to behavioral change and appreciation of the app were based on the questionnaires and included all that responded to the follow-up survey (see Fig. 1, Baseline and Follow-up response). Reasons for nonuse or drop-out, appreciation of the app and age differences in beliefs and attitudes were based on chi-squared tests and *t*-tests and their degrees of freedom. To investigate change in beliefs and attitudes related to behavioral change (MCLHB-DRR Scale) from baseline to post-intervention, we used linear mixed models adjusted for age, sex, and educational level. As a sensitivity analysis, participants from the 2019-sample who were recruited via the extra recruitment strategies (see Fig. 1) were excluded to investigate whether the results were driven by this difference in recruitment. All analyses were performed in Stata 17 (StataCorp, College Station, TX, USA), and the level of statistical significance was *p* < 0.05 in two-tailed tests.

## RESULTS

### Participants

A total of 686 individuals were invited to participate, of whom 299 (response rate: 43.6%) completed the baseline survey and subsequently received information on how to install the app (see Fig. 1). In total, 190 (63.6%) participants completed the follow-up questionnaire. A loss to follow-up analysis on survey response revealed no differences between study-completers and drop-outs/non-responders for sex (*χ*^2^ (degrees of freedom = 1) = 0.027, *p* = 0.870), age (t(297) = –0.664, *p* = 0.507), level of education (*χ*^2^(1) = 0.202, *p* = 0.653), risk factor profile (t(161) = 1.50, *p* = 0.140), version of app (standard versus extended version; *χ*^2^(1) = 2.19, *p* = 0.139) or operating system (iOS versus Android; *χ*^2^(1) = 3.143, *p* = 0.076). Table 2 describes the characteristics of the total study sample and stratified by study year. There were no socio-demographic differences observed in version of the app and year of study, except for a higher percentage of highly-educated individuals participating in the 2019 study compared to the 2018 study.

**Table 2:** Characteristics of the total sample and stratified by study year

Variables^a^	Total baseline sample	2018-study	2019-study	*p*
	(*n* = 299)	(*n* = 89)	(*n* = 210)	
Version of the app, *n* (%)				<0.001
Standard version	198 (66.2%)	89 (100%)	109 (51.9%)	
Extended version	101 (33.8%)	NA	101 (48.1%)	
Sex, *n* (%)				0.55
Female	149 (49.8%)	42 (47%)	107 (51.0%)	
Male	150 (50.2%)	47 (53%)	103 (49.1%)	
Age, mean±SD (range)	60.2±8.3 (40–74)	61.4±7.8 (40–74)	59.7±8.5 (41–74)	0.11
Age groups, *n* (%)				0.09
40–59 y	133 (44.5%)	33 (37%)	100 (47.6%)	
60–75 y	166 (55.5%)	56 (63%)	110 (52.4%)	
Level of education^b^, *n* (%)				0.03
Low	19 (6%)	10 (11%)	9 (4%)	
Middle	104 (34.8%)	35 (39%)	69 (33%)
High	176 (58.9%)	44 (49%)	132 (62.9%)	
Marital status, *n* (%)				0.91
Married/living together	241 (80.6%)	73 (82%)	168 (80.0%)	
Unmarried	14 (5%)	3 (3%)	11 (5%)	
Divorced	33 (11%)	10 (11%)	23 (11%)	
Widowed	11 (4%)	3 (3%)	8 (4%)	

^a^Percentages do not always add up to 100% because of rounding. ^b^ Self-reported highest finalized degree, divided into low (primary school or low vocational education), middle (intermediate secondary education or intermediate vocational or higher secondary education) and high (higher vocational education or university). NA, not applicable; SD, standard deviation.

### Usage tracking data

A total of 167 participants (55.9%) installed the app according to the usage tracking data. Approximately 19% (*n* = 56) of the total sample contacted the research team via email or telephone with questions on installing the app and password resets. The usage of the app was on average 30.4 days (from installation to last daily notification; SD 39.9, range 1 to 181, with 40% (*n* = 66) of the participants using the app less than seven days). Participants received on average 20.3 (SD 23.1; range 1 to 98) daily notifications, with 41% (*n* = 68) receiving six or less and 7% (*n* = 11) receiving more than 70 notifications. Of all participants who were randomized to the extended version of the app (*n* = 101), 50 (50%) installed the app but only 16 of these 50 participants (32%) used the main additional feature of the extended version (tailoring based on the DLBQ constructs). Those who did not use this additional feature (*n* = 34) either chose themes that did not include the tailoring feature (*n* = 13; 38%) or received only three or less daily notifications on a relevant theme (for tailoring to appear; *n* = 21; 62%).

**Fig. 4:**
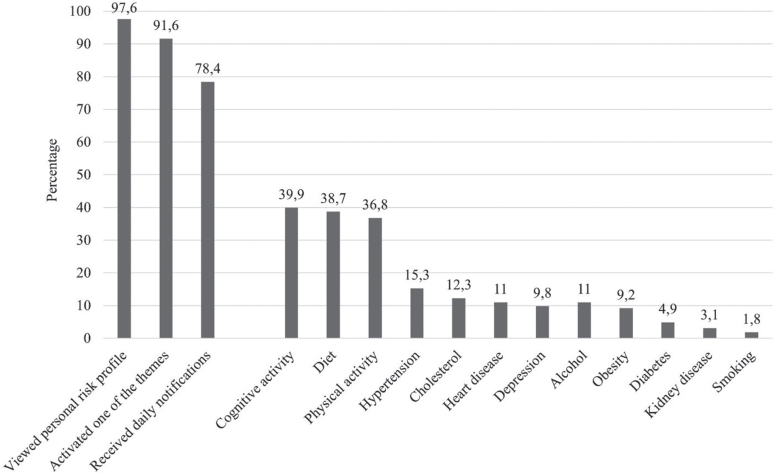
Subsequent steps taken in the app according to user-tracking data. Individual factors reflect the percentage of participants that have activated this theme in the app to receive daily notifications (information, quiz item, behavioral challenge).

Figure 4 displays the frequency of the subsequent steps/functions used in the app and the themes chosen to receive daily notifications. Among the 167 participants who installed the app, the majority (*n* = 163, 97.6%) viewed their personal risk profile. Health factors that were most often present were hypertension (*n* = 81, 50%), high cholesterol (*n* = 52, 32%), and chronic kidney disease (*n* = 36, 22%). Room for lifestyle improvement was highest for physical activity (*n* = 137, 84.1%), healthy diet (*n* = 134, 82.2%) and cognitive activity (*n* = 69, 42%), and these lifestyle themes were also most often chosen for reception of daily notifications (cognitive activity *n* = 65, 40%, healthy diet *n* = 63, 39% and physical activity *n* = 60, 37%). Women installed the app more often than men (63.1% versus 48.7%; *χ*^2^(1) = 6.304, *p* = 0.012). No age differences in app usage were found, nor between users of different versions of the app (standard versus extended) and year of study (2018 versus 2019).

### Online questionnaires

#### Reasons for non-use and drop-out

Of all 70 participants that did not use the app and responded to the follow-up assessment, 41 completed items on reasons for non-use (the remaining 29 had incomplete survey responses). Of all participants that responded to the follow-up assessment and did use the app (*n* = 120), 43 (36%) reported to have stopped using the app during the intervention. Reasons for non-use and drop-out were mostly technical problems such as problems with installation, sporadic logouts and forgetting username or password (*n* = 21; 51% and *n* = 30; 70% respectively), followed by motivational issues (*n* = 11; 27% and *n* = 5; 12%) and lack of time (*n* = 9; 22% and *n* = 8; 19%). No differences were observed in reasons for non-use and drop-out for age, sex, and educational level between the two app versions (Table 3).

**Table 3:** Reasons for non-use and drop-out during the study by version of the app

	Non-use (*n* = 41)		Drop-out (*n* = 43)	
Self-reported	*Standard version*	*Extended version*	*Standard version*	*Extended version*
reasons, *n* (%)	*(n = 22)*	*(n = 19)*	*(n = 22)*	*(n = 21)*
Motivational issues	6 (27%)	5 (26%)	1 (5%)	4 (19%)
Lack of time	6 (27%)	3 (16%)	5 (23%)	3 (14%)
Technical problems	10 (46%)	11 (58%)	16 (73%)	14 (67%)

Reasons for non-use and drop-out based on the online questionnaires. The number of participants varies due to incomplete survey response).

### Beliefs and attitudes related to dementia risk reduction

Table 4 shows the baseline measurement of beliefs and attitudes related to dementia risk reduction and a comparison of those who did and who did not use the app. Participants who did use the app scored higher on perceived benefits and self-efficacy and lower on perceived barriers compared to participants who did not use the app. Differences in age and educational level were observed. Participants under the age of 60 years perceived more benefits than older participants (t(297) = 2.714, *p* = 0.007). Low-to-middle educated participants perceived dementia as more severe (t(297) = 2.352, *p* = 0.019) and perceived more barriers (t(297) = 2.476, *p* = 0.014) compared to highly educated participants. No baseline differences were observed for users of the standard versus extended version of the app, and for the 2018 versus 2019 sample. In the intention-to-treat analyses, no differences were found on changes in beliefs and attitudes over time (see Supplementary Table 1).

**Table 4:** Baseline beliefs and attitudes in the total sample and differences in app use

	Total sample	Used app	Did not use app	*p*
	(*n* = 299)	(*n* = 167)	(*n* = 132)
Perceived susceptibility	8.9±2.0 (3 – 14)	8.9±2.0 (3 – 14)	9.1±2.1 (3 – 13)	0.646
Perceived severity	15.6±3.3 (5 – 24)	15.7±3.4 (5 – 24)	15.5±3.2 (7 – 24)	0.550
Perceived benefits	7.1±1.4 (3 – 10)	7.3±1.5 (3 – 10)	6.9±1.4 (4 – 10)	0.026
Perceived barriers	8.3±2.5 (4 – 16)	8.1±2.5 (4 – 16)	8.7±2.5 (4 – 16)	0.011
Cues to action	11.9±2.6 (4 – 20)	11.9±2.6 (4 – 19)	11.8±2.6 (4 – 20)	0.677
General health motivation	12.3±1.7 (7 – 15)	12.4±1.7 (8 – 15)	12.1±1.7 (7 – 15)	0.154
Self-efficacy	6.6±1.4 (2 – 10)	6.8±1.4 (2 – 10)	6.4±1.4 (2 – 10)	0.025

Baseline beliefs and attitudes regarding dementia risk reduction in the total sample and differences in those that did and did not use the app. Values are presented as means±SD (range).

### Appreciation of the app

Of all participants who used the app and responded to the follow-up assessment (*n* = 120), 21 stopped before or during the evaluation, leaving 99 to 106 participants in these analyses, depending on the item. Participants reported to have spent an average of 6.4 min per day on the app (SD 7.8, range 0 – 45). The app was overall graded with a 6.3 (SD 2.0; range 2 – 10) on a scale of 0 to 10, with higher scores being better. Low-to-middle educated participants spent more minutes per day (9.4, *n* = 35) on the app than highly educated participants (4.7, *n* = 65) did (t(98) = 2.97, *p* = 0.004), and also graded the app slightly higher (low/middle: 6.8, *n* = 34; high: 6.0, *n* = 65; t(97) = 2.06, *p* = 0.042). The majority (*n* = 67, 55.8%) of all participants wanted to keep using the app. No differences were observed for sex, age, study year and version of the app.

**Fig. 5:**
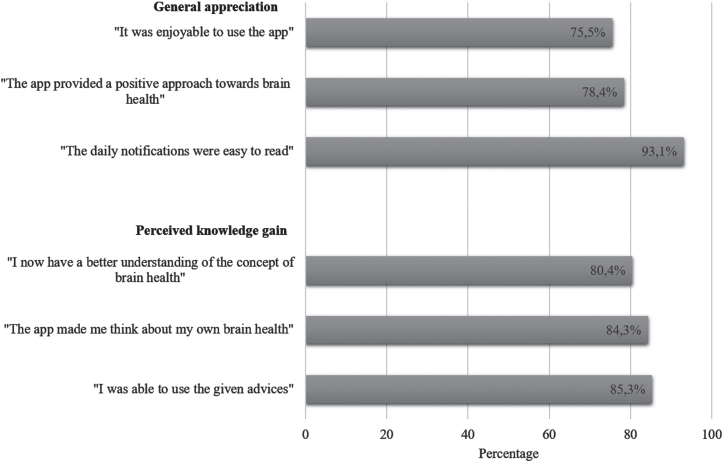
User appreciation of the MyBraincoach app (*n* = 102). Percentages reflect the proportion of participants agreeing with the statement or answering neutrally (totally agree, agree, and neither agree nor disagree).

Figure 5 shows the results of the self-reported data of the post-assessment survey concerning the user evaluation. Participants using the extended version less often stated that the app was enjoyable (63%, *n* = 22 versus 82%, *n* = 55; *χ*^2^(1) = 4.60, *p* = 0.032). Low-to-middle educated participants stated more often than highly educated participants that the daily notifications were easy to read (100%, *n* = 36 versus 89%, *n* = 59; *χ*^2^(1) = 4.10, *p* = 0.043), that they perceived the advices as useful (100%, *n* = 36 versus 77%, *n* = 51; *χ*^2^(1) = 9.59, *p* = 0.002), and have gained a better understanding of brain health (94%, *n* = 33 versus 74%, *n* = 49, *χ*^2^(1) = 6.02, *p* = 0.014). Younger participants (<60 years) more often agreed that the app was easy to use (84%, *n* = 42 versus 59.6%, *n* = 31; *χ*^2^(1) = 7.45, *p* = 0.006).

### Sensitivity analysis

A series of sensitivity analyses were performed excluding those from the 2019-sample who were recruited via the extra recruitment strategies of advertisement on the website of the Dutch Brain Foundation and via a newsletter of a large health care institution in the south of the Netherlands. Results on usage pattern and the self-reported questionnaires were similar (data not shown).

## DISCUSSION

### Principal findings and interpretation

This proof-of-concept study investigated the MyBraincoach mobile application on usage patterns, beliefs and attitudes related to dementia risk reduction, and appreciation in 299 non-demented, community-dwelling Dutch participants in midlife. Overall, app usage was modest with only 56% using the app and 40% of them using the app for more than 1 week. Technical issues were the most frequent self-reported reason for non-use or drop-out. Those who did use the app, evaluated it positively, with most participants stating to have improved their knowledge on brain health, with younger (<60 years) and low-to-middle educated participants evaluating the app most positively.

This study was limited by the considerable number of participants who did not install or start using the app (*n* = 132, 44.2%) or did not respond to the follow-up assessment (*n* = 109, 36.5%). This should be taken into consideration when interpreting the results. These participants perceived upfront more barriers, less benefits and lower self-efficacy towards dementia risk reduction, the app was probably only used by participants who were a priori motivated to engage in a brain-healthy lifestyle. Other possible explanations for the non-adherence could be the technical issues regarding the app, involving difficulties associated with registration, login, and server time-outs. To illustrate, of all participants who installed the app and activated a lifestyle theme, 14.4% (*n* = 22) (see Fig. 4) never received a daily notification, which is likely the consequence of system failure logouts. As for the 2019 sample specifically, stronger rules on privacy assurance resulted in extra steps to download the extended app, which resulted in high drop-out rates. Furthermore, in the week after the link to the post-assessments were e-mailed to participants of the 2019 study, Maastricht University was hit by an institution-wide cyber-attack. Because of the subsequent security shutdown, our survey was offline, and we were unable to reach participants in the first five weeks after the end of the intervention period. This might have led to additional drop-out and worse information recall by participants, which might have influenced the results. Yet, attrition rates are generally high in digital interventions, and our loss to follow-up can be considered average compared to those reported in previous systematic reviews on health-app usage (47%) [35, 36]. Notably, the technical issues have by now been resolved and follow-up studies with the app are ongoing in different countries.

Participants older than 60 years experienced less benefits towards dementia risk reduction. It could be that older people consider it too late for interventions to delay conversion to dementia. Several studies show that older participants also experience barriers to engage in web-based interventions [37, 38]. Yet, older participants still evaluated the app in a positive manner, and there were no differences between age groups in use of the app or drop-out of the study. Low-to-middle educated participants perceived dementia as more severe and experienced more barriers to change than higher educated participants. Yet, they also spent more time with the app and gave the app a slightly better overall score, which could suggest that they were more engaged. Importantly, the app was designed to make it suitable for less educated or less health-literate people (e.g., prompting people with short messages that appeared automatically, testing of texts on language proficiency level), since these sub-groups are often hard-to-reach in web-based lifestyle interventions [15, 36]. Previous research showed that lower education and lower socio-economic position is associated with a higher risk for dementia, and that this is substantially explained by an unfavorable modifiable risk score [23]. Engaging this hard-to-reach group is thus important for achieving the goals of dementia risk reduction in the general population and reducing brain-health inequalities [9].

Beliefs and attitudes regarding dementia risk reduction did not change over time. The scale has, to our knowledge, not been used to assess the effect of (lifestyle) interventions and, therefore, comparisons with other studies cannot be made. Given the observation that 40% (*n* = 68) of all participants received six or less daily notifications and that the effect measurement only took place after 12 weeks, an explanation could be that the app was insufficient in achieving this aim, or that beliefs and attitudes did change directly after viewing one’s risk profile and/or receiving the daily messages, but that this effect was not sustained over time.

### Strengths and limitations

Strengths of this study include the measurements of both objective usage-tracking and self-reported data. Regarding the development of the mobile app, we checked the accuracy, completeness, and readability of the custom-made daily notifications by renowned experts in the field and tested the general acceptance of this app in potential end-users and a client panel. In addition, we designed the app around multiple dementia risk factors (12 in total) and collaborated with different stakeholders in a multidisciplinary approach, which is in line with WHO guidelines [5]. Next, in contrast to most other web-based interventions to improve brain health [39], our mHealth platform is available free of charge to the general public in the Netherlands and its content can easily be adapted to the setting and language of other countries, thereby providing a bridge between dementia risk reduction research and the community.

This study, however, also has limitations that need to be addressed. First, this study did not use a control group and randomization (in the total sample), which means that the internal validity of the intervention is low. Furthermore, recruitment bias may have occurred due to the inclusion from responders of a previous dementia risk awareness survey [7] who expressed interest in brain health studies, and additional recruitment via the websites of the Dutch Brain Foundation and a large health care institution. This makes our results less generalizable to the general population. Furthermore, except for the export of the usage pattern of participants, all outcome measures were based on self-report. Socially desirable answers should therefore be taken into account when interpreting results on appreciation of the app. In addition, the questionnaire assessing user appreciation was custom-made and not validated.

### Implications and recommendations for future research

First, it is important to improve the usability of the tool before executing further intervention studies, since it is impossible to draw conclusions concerning the effectiveness of (parts of) an intervention if participants were not able to use it the way it was supposed to. Technical issues that hampered usage in the present study have by now been resolved. When further developing the behavioral change aspects of this app, the right balance between mHealth and in-person counseling should be considered [40, 41]. For example, a blended care approach, in which the mHealth tool is combined with face-to-face or online coaching sessions, could have beneficial effects on lifestyle improvements [42, 43]. The extended version of this app did attempt to tailor the daily notifications towards the individual, but still was a stand-alone intervention without behavioral support of a coach. Hence, it failed to show any notable benefits over the standard version. Last, future studies should ideally consider randomized controlled designs in investigating the effect of mobile applications to improve brain health in order to answer questions regarding causality.

### Conclusions

This study shows that the MyBraincoach mobile app could be a useful tool for raising awareness and knowledge transfer regarding the possibility of dementia risk reduction. It could therefore be used in ongoing preventative measures and lifestyle-based intervention studies to optimize the potential of primary prevention of dementia. Yet, this and other apps should focus on improving the usability and investigating the effect of adding features to increase motivation and, ultimately, achieve behavioral change.

## Supplementary Material

Supplementary Material

## Data Availability

The datasets used and analyzed during the current study are uploaded in Dataverse repository (https://doi.org/10.34894/ZPBCEO) and available on reasonable request.
