# Supplementary material for: Using mHealth for Primary Prevention of Dementia: A Proof-of-Concept Study on Usage Patterns, Appreciation, and Beliefs and Attitudes Regarding Prevention
Source: J Alzheimers Dis. 2023 Aug 1;94(3):935–48. doi: 10.3233/JAD-230225 (PMC10473134; doi:10.3233/JAD-230225)
Supplement: Supplementary Material [file jad-94-jad230225-s001.pdf]

Supplementary Material

Using mHealth for Primary Prevention of Dementia: A Proof-of-Concept Study on Usage Patterns, Appreciation, and Beliefs and Attitudes Regarding Prevention

Supplementary Table 1. Beliefs and attitudes over time in the total sample

Variables[a]	Total pre-survey, mean±SD; range	Total post-survey, mean±SD; range	B	*p*
Perceived susceptibility	8.9±2.0 (3 – 14)	8.8±2.2 (3 – 15)	-0.07	0.56
Perceived severity	15.6±3.3 (5 – 24)	15.5±3.5 (5 – 25)	-0.26	0.18
Perceived benefits	7.1±1.4 (3 – 10)	7.0±1.4 (3 – 10)	-0.13	0.20
Perceived barriers	8.3±2.5 (4 – 16)	8.4±2.5 (4 – 15)	0.23	0.16
Cues to action	11.9±2.6 (4 – 20)	11.9±2.6 (4 – 18)	0.09	0.60
General health motivation	12.3±1.7 (7 – 15)	12.3±1.7 (7 – 15)	0.01	0.92
Self-efficacy	6.6±1.4 (2 – 10)	6.5±1.4 (2 – 10)	-0.05	0.62

Beliefs and attitudes over time regarding dementia risk reduction in the total sample. Values are presented as means ± SD (range).
